# Interethnic Variations and Clinical Features of Spondyloarthropathies in a Middle Eastern Country

**DOI:** 10.2174/1874312901812010010

**Published:** 2018-01-31

**Authors:** Mohammed Kamil Quraishi, Humeira Badsha, Bhavna Khan, Muhammad Shahzeb, Srilakshmi Hegde, Ayman Mofti, Kong Kok Ooi

**Affiliations:** 1 Department of Urology, Medway Maritime Hospital, Kent, England, UK; 2 Department of Rheumatology. Dr. Humeira Badsha Medical Center, Dubai, UAE.; 3 Integrated Rheumatology and Arthritis Centre, Dubai Healthcare City, Dubai.; 4 Department of Medicine, Jinnah Medical College Hospital, Karachi, Pakistan.; 5 Department of Rheumatology, Al Biraa Arthritis & Bone Center, Dubai, UAE.; 6 Department of Rheumatology, Tan Tock Seng Hospital, Singapore

**Keywords:** Ankylosing Spondylitis, Spondyloarthropathy, spondyloarthritis, UAE, Emirates, ethnicity, HLA B27

## Abstract

**Objectives:**

The study aimed to demonstrate the interethnic differences and clinical features of Spondyloarthropathy(SpA) patients in a diverse Middle Eastern Country.

**Methods:**

A retrospective review of medical records to collect the required data was conducted for SpA patients at two study institutions in the United Arab Emirates.

**Results:**

Of 141 SpA patients found, 88 AS(Ankylosing Spondylitis) patients and 53 ‘other SpA’ patients were identified. Males constituted 81% of AS and 55% of ‘other SpA’ patients. Patients with AS and ‘other SpA’ had a mean age of symptom onset of 28 and 34 years, respectively.

49% and 40% of AS and ‘other SpA’ patients had a history of Anti-TNF therapy usage. Enthesitis and Uveitis were noted in 16% and 18% of AS patients whilst 53% and 11% in ‘other SpA’ patients, respectively.

Caucasian, Indian Subcontinent and Arabs constituted 93% of our cohort. Mean age of onset of symptoms in the Indian Subcontinent ‘other SpA’ group was much greater than the other two ethnicities. Duration of symptoms to diagnosis was 3.5 and 4 years in AS and other SpA patients' respectively. HLA-B27 positivity was found in 53%, 80% and 93% of Arab, Indian Subcontinent and Caucasian AS patients, respectively, whilst seen in 50%, 25% and 33% of the same respective ethnicties in ‘other SpA’ patients.

**Conclusion:**

This study on 141 patients is the largest to analyse inter-ethnic variations in SpA patients in the region. Our cohort shows a short delay in diagnosis with a relatively higher Anti-TNF usage.

## 1 INTRODUCTION

Spondyloarthropathies (Spondyloarthritis)(SpA) are a collection of chronic inflammatory rheumatic conditions that share multiple clinical features including axial and/or peripheral arthritis, enthesitis, absence of the serum rheumatoid factor and presence of common extra articular manifestations [1]. The spondyloarthropathy family comprises of Ankylosing Spondylitis(AS), psoriatic arthritis, reactive arthritis, inflammatory bowel disease associated SpA, juvenile SpA and undifferentiated SpA(uSpA) [2]. These diseases are strongly associated with the genes of the Major Histocompatibility Complex (MHC), in particular, the Human Leucocyte Antigen(HLA) B27 [3, 4].

Research in recent years has documented the clinical features of Ankylosing Spondylitis in the Middle East. The data highlighted the wide variation in HLA-B27 positivity across the region [5-10] There is an even greater paucity of documentation of non-AS SpA in the Middle East, which may affect the management of these patients [11]. Uppal et al. found several interesting differences between AS and undifferentiated SpA patients amongst the South Asian and Middle Eastern patients in Kuwait. However there remains a lack of information on whether similar inter-ethnic SpA profiles exist amongst other Middle Eastern countries. With the exception of a small case series on AS patients in the UAE, there has been no other comparison of inter-ethnic profiles of any SpA subtype in the Middle East [8].

In recent years, UAE has seen significant reforms in demographic and health care organization [12]. As an emerging market in medical tourism, Dubai in particular boasts a very diverse patient population. With only 11% of the UAE population constituting the native Emiratis, there remains a respectable opening for studying inter-ethnic profiles in the country [13]. The inter-ethnic profiling may be of clinical benefit if there is an awareness of the prevalence of certain features in specific patient groups [14].

This case series is the first of its kind in studying all the subtypes of SpA in the UAE. The primary aim of our study was to demonstrate the interethnic variations and clinical features of Spondyloarthritis (SpA) patients from a specialized arthritis center and a Rheumatology department at a multi-specialty center in Dubai, United Arab Emirates.

## 2 MATERIALS AND METHODS

We conducted a retrospective study on patients with a diagnosis of SpA at a specialized arthritis centre and a rheumatology department of a multi-specialty medical centre in Dubai, UAE. Payment modalities for the patients at these private centres range from self-funding to comprehensive insurance cover. Patients were identified by examining electronic records for ICD 9 codes during the month of August 2012. Reviews of medical records were carried out to obtain the required data. The study was approved by an internal ethics committee at the specialized arthritis centre and was conducted in accordance with the recommendations of the Declaration of Helsinki.

The socio-demographic data collected included age, gender, smoking status, ethnicity and family history of SpA. Clinical data included the age of symptom onset, age of diagnosis, characteristic of back pain (inflammatory back pain was defined as low back pain and stiffness for more than three months that improves with exercise, but not relieved by rest), presence of peripheral arthritis, extra-articular manifestations (enthesitis, uveitis, psoriasis and inflammatory bowel disease), Human Leucocyte Antigen-27(HLA-B27) status, and treatment were recorded. The relevant information was obtained by accessing the last clinic consultation notes, when appropriate. HLA-B27 status was based on the result of the standard assay on peripheral blood as conducted by the local pathology laboratory. HLA-B27 subtype data was not recorded in our cohort due to inconsistent testing. The Bath AS Disease Activity Index(BASDAI) and Schoebers test are routinely conducted by the physician at every consultation for AS patients, but only the last documented entry was used for our analysis. Radiological evaluation was used to assess the evidence of unilateral or bilateral sacroiliitis on X-ray. A Magnetic Resonance Imaging (MRI) scan was used when the diagnosis was unclear.

Both centres adopted the same diagnostic criteria for the management of SpA. Patients were categorised as Ankylosing Spondylitis (AS) based on the modified New York criteria. Patients who fulfilled the European Spondyloarthropathy Study Group(ESSG) criteria but who did not meet the modified New York criteria were classed in the ‘other SpA’ group. In recent years, the terminology has evolved to classify as radiographic and non-radiographic SpA, however they were not classified in this manner at the time of diagnosis.

The analysis was conducted on the statistical package SPSS v20. The mean values were calculated for all continuous variables. The Unpaired Student’s t test was utilised to compare the means of continuous variables, whilst the Fischer’s exact test was used to evaluate percentages. A One-way ANOVA test was performed to study differences in means of more than two groups. A p-value of <0.05 was deemed to be of statistical significance for this analysis.

## 3 RESULTS

### 3.1 Socio-demographic Features

A total of 141 patients were identified, of whom 88 held a diagnosis of AS and 53 of other SpA. Table **1** summarises the socio-demographic characteristics of the patient populations. The ‘other SpA’ group constituted 2 IBD related SpA, 5 psoriasis related SpA, 2 reactive arthritis related SpA and 44 undifferentiated SpA. The difference in the mean age of symptom onset(p<0.01) and diagnosis(p<0.001) between AS and ‘other SpA’ patients was statistically significant. Nine AS patients were known to have a family history of AS, but their HLA-B27 status were unknown. No ‘other SpA’ patients were known to have any family history of SpA. HLA-B27 was positive in 76% of our AS patients but only 36% in the ‘other SpA’ cohort.

### 3.2 Clinical Features

The clinical characteristics of the patients are summarised in Table **2**.

Vitamin D insufficiency was observed in 42 (48%) AS and 26 (49%) ‘other SpA’ patients at some point during their follow-up care. The knees were the most commonly affected joint in ‘other SpA’ patients (49%) with 58% of these patients having bilateral knee involvement. Twenty three (43%) and five (9%) ‘other SpA’ patients showed back and neck involvement respectively. Eight AS patients had a characteristic bamboo spine whilst this feature was not observed in the ‘other SpA’ patients.

Achilles enthesitis was the most common form of enthesitis and was found in 28% and 13% of ‘other SpA’ and AS patients respectively. Plantar Fascitis was noted in 9(17%) ‘other SpA’ and 2(2%) AS patients Seventeen (32%) of ‘other SpA’ patients were on anti-TNF therapy at the last follow-up, whilst four(8%) other patients had previously utilised it. Anti-TNF was prescribed to 29 (33%) AS patients at the last follow up, whilst 14 (16%) others previously used this treatment modality. Nine(10%) AS and four(8%) ‘other SpA’ patients were on Methotrexate at the last follow-up; but seventeen(19%) AS and twelve (23%) ‘other SpA’ patients were on Sulfasalazine at the last follow-up.

### 3.3 Ethnic Variations

The inter-ethnic variations in the features of SpA were studied in the Arab, Caucasian and Indian Subcontinent patients since they contributed to the large majority of the cohort(93%). Table **3** summarises some of the features.

Uveitis occurred in 5%, 23% and 12% of Arab, Indian Subcontinent and Caucasian AS patients, respectively. In ‘other SpA’ patients, uveitis occurred in 9% of Caucasians, 18% of Indian Subcontinent and none in Arab patients. The inter-ethnic differences in uveitis were not statistically significant in the patient populations. Enthesitis was observed in 10% of Arab, 15% of Indian Subcontinent and 24% of Caucasian AS patients. It was seen in 75% of Arab, 44% of Indian Subcontinent and 45% of Caucasian ‘other SpA’ patients. Of the eight AS patients that had a bamboo spine, six(15%) were from the Indian subcontinent, one(6%) patient was Caucasian and another(5%) was Arab. The mean(SE) BASDAI for the AS patients was 2.7(0.49) for Caucasian, 3.3(0.68) for Arab, And 3.6(0.41) for the Indian Subcontinent patients.

In patients with AS, HLA-B27 was positive in 53% of Arabs, 80% of Indian subcontinent and 93% of Caucasians. This contrasted to the low HLA-B27 positivity in the ‘other SpA’ population with 50% in Arabs, 25% in Indian Subcontinent and 33% in Caucasian patients. The difference in HLA positivity between the ethnic groups was statistically significant in the AS patients but not in the ‘other SpA’ patients.

## 4 DISCUSSION

The presence of variations in clinical features of spondyloarthropathies across various ethnicities and geographical demographics is well recognized [15, 16]. The UAE offers a great insight into studying such conditions owing to its diverse ethnic population. This ethnic diversity in our cohort reflects to a certain degree the general demographics of the UAE, with Indian Subcontinent expats and Arab nationals comprising an estimated 54.7% and 27.5% of the UAE population [17, 18]. Our results, based on a relatively large patient cohort, will provide a stepping stone for setting up local guidelines for better standardised management.

### 4.1 Clinical Features

AS and uSpA form the largest subgroups of spondyloarthropathies [19, 20]. USpA constituted 83% of the ‘other SpA’ group, and together with AS, they formed 94% of the total cohort. Due to lack of published data on ‘other SpA’ and since uSpA amounted to the large majority of this group, we decided to make comparisons with other documented uSpA data, mentioned in the discussion section. 6% of the AS patients did not have inflammatory back pain but were diagnosed on the basis that they still fulfilled the Modified New York criteria. Inflammatory back pain is one of the main clinical features of uSpA [20], but only 53% of the ‘other SpA’ cohort exhibited it. This prevalence is similar to those documented in other uSpa studies [21, 22].

### 4.2 Gender Distribution

The male predominance in the Caucasian populations of our AS and ‘other SpA’ groups was similar to those AS and uSpA data published in other literature [20, 23, 24]. The patients from our Indian subcontinent cohort however, exhibited a much poorer male to female ratio than the previously published data on Indian AS and uSpA groups, which ranged from 5:1 – 16:1 [22, 25-27]. The Indian Subcontinent category included countries such as Pakistan and Sri Lanka which had not been featured in these reported studies. The inclusion of spondyloarthropathy patients from these other countries perhaps explains the distinct differences in gender ratio. The Arab AS cohort displayed a very high male prevalence(95%) than that documented in similar Middle Eastern patient groups [7, 14, 28] including the Arab group of the previous UAE study [8]. Our series is the first to list the male to female sex ratio for any non-AS SpA patients of Arab ethnicity at 2:1.

### 4.3 Onset & Diagnosis

The mean age at the onset of symptoms in AS Arab and Indian subcontinent patients was similar to those in the Arab and Indian populations of the Kuwaiti and UAE study [8, 11]. Interestingly, the onset of symptoms in ‘Other SpA’ patients occurred 8.2 and 9.8 years later in Arabs and Indian Subcontinent(South Asian) patients, respectively, than the Kuwaiti uSpA cohort [11]. The age at the onset of symptoms in Caucasian populations reflected those observed in a larger Caucasian dominant series [23, 24, 29].

Spondyloarthropathies usually take 5-6 years to be diagnosed after symptom onset, particularly in the presence of early limited features [30]. This delay may even stretch to over 10 years depending on circumstances [31]. Most patients in our study had a very short delay to diagnosis. These results are much better than those published in other Middle Eastern [11, 14, 28] countries and elsewhere [24, 32-34]. The

Arab subset of our ‘Other SpA’ group was an exception, with long delays to diagnosis compared to the other Arab uSpA data. We speculate that the Arab ‘other SpA’ cohort possibly initially presented with slowly progressive non-specific or peripheral joint symptoms which further exacerbated by delays in seeking medical attention.

### 4.4 Extra-articular Features

Enthesitis was seen twice in as many ‘other SpA’ patients as AS patients and the difference was strongly significant. The occurrences of enthesitis amongst the ethnicities were similar to the large range of 20-60% occurrence reported in the literature which varied with the study population [8, 11, 22, 23, 26, 28, 35]. The Arab AS patients in our study had lower occurrences of enthesitis than those reported in other Arab studies [8, 9, 11].

Uveitis rates in our ethnic cohorts were also similar to the overall ranges(9-22%) reported in other studies with similar ethnicities and SpA subsets [8, 20, 23, 25, 27, 35] [. Al Attia reported a much higher occurrence in Arab and lower occurrence in South Asian(Indian Subcontinent) population of uveitis in AS patients [8]. Our findings suggested the prevalence of uveitis in Indian subcontinent and Arab patients similar to those reported in other Indian and Arab dominant studies [9, 26, 27, 35]. The absence of uveitis amongst the Arab ‘other Spa’ patients could be associated with the low predominance of uveitis amongst Arab SpA patients or a consequence of a small Arab cohort.

### 4.5 Smoking

A significant proportion of the patients had a history of smoking. Smoking is associated with poor functional outcomes in spondyloarthropathy patients [36]. Furthermore, spondyloarthropathy patients are at an independently increased risk of cardiovascular disease [37]. It is therefore vital for patient education to form an integral component of management.

### 4.6 Anti TNF

Anti TNF usage has been poorly reported in the Arab countries. The usage in our cohort is much greater than 9% reported in an Egyptian spondyloarthropathy registry [35]. This may be because of the tertiary nature of the clinics which tend to deal with the more severe cases. The long term follow up seen in this database may explain the increasing prevalence of anti-TNF usage with time as patients deteriorate justifying anti-TNF usage.

### 4.7 HLA-B27 Positivity

The association between HLA-B27 and SpA has led to significant research into HLA-B27 and its subtypes. There appears to be a rough correlation between the incidences of HLA-B27 in the general population with the incidences in the same ethnic SpA population [38, 39].

The prevalence of HLA-B27 is known to vary only slightly amongst the resident population of the Arab countries. It has been found to be 4% in Kuwait [5], 1.4% in Lebanon [6], 2.4% in Jordan [7], 6.4% in UAE [40] and 0.3% in Oman [41]. Arab and Indian Subcontinent people in the UAE were found to have a background HLA-B27 prevalence of 5.7% and 7.4%, however no data is available on Caucasians in this cohort. The HLA prevalence in the general Indian population was found to be varying between 26% [22, 42, 44] with relatively lower positivity seen in the South Indian population [45]. However specific geographical populations in India have been found to have HLAB27 as high as 19.6-29% [46]. In Caucasian, the background prevalence of HLA-B27 was found to be approximately 8-10% [3, 47]. We would therefore expect to find higher HLA-B27 prevalence in our Indian subcontinent and Caucasian population, which is the case.

The HLAB27 prevalence in Caucasians amongst our cohorts was observed to be similar to 90% reported in Caucasian AS patients but was much lower than the 70% in Caucasian uSpA patients [3].

There is a marked variation between HLA-B27 prevalence of AS patients in Arab countries, but is known to be generally lower than the global figures [10]. A review derived the prevalence of HLA-B27 to be 64% in Arab AS patients, by pooling together several studies [10]. HLA-B27 prevalence was found to vary between 76 – 94% in Indian AS patients [29, 30, 48]. Our results in fact are very similar to those seen in the study on UAE AS population where Arab and Indian Subcontinent patients had HLA-B27 prevalence of 56% and 81%, respectively [8]. It is however slightly different to the Qatar resident Arab(74%), Asian(61%), Kuwait resident Arab(86.7%) and Indian subcontinent(75%) AS patients [10, 11].

Our Indian Subcontinent ‘other SpA’ demographic had a much lower HLA-B27 prevalence than the previously reported 45% and 84% amongst Indian uSpA patients [25, 43]. Our ‘other SpA’ patient profile had a markedly lower HLA-B27 prevalence than the Kuwaiti uSpA population both in Arab(66.7%) and Indian subcontinent(71.4%) patients [11].

### 4.8 Vitamin D Insufficiency

Vitamin D insufficiency characterized as <30ng/ml was found to be nearly 50% in our SpA cohort during testing performed at any random point in their follow up. Its significance was found in its association with higher SpA disease activity and severity [49, 50].

## 5 LIMITATIONS

Not everyone had HLA-B27 testing which may explain the low prevalence, particularly in the ‘other SpA’ group(55% were tested). This may be due to the fact that the results of the test were perhaps lost or the clinician felt that the result did not add enough diagnostic evidence to justify the cost of the test. Insurance cover was not recorded for patients, which may have been a possible source of bias in view of the high usage of Anti-TNF therapy in our cohort. Despite 83% of ‘other SpA’ group being constituted by uSpA, the ‘other SpA’ might have markedly affected the results, perhaps accounting for the differences in the results between other uSpA study groups.

## CONCLUSION

Our study demonstrates the largest database of clinical features of Spondyloarthropathy patients in the UAE. It also appears to be a noteworthy research work analysing inter-ethnic variations in SpA patients in the region. The study highlights that HLA-B27 prevalence is relatively poorer amongst Arab AS patients and ‘other SpA’ patients. Spondyloarthropathy patients in the UAE generally have a very short delay in diagnosis. The study also highlights the relatively higher prevalence of Anti-TNF usage in the UAE. Greater emphasis should be placed on patient and clinician awareness regarding uveitis in Indian Subcontinent SpA patients in view of its high prevalence. Improvements need to be made for awareness among primary care physicians and rheumatologists regarding the varied presentations of ‘other SpA’ Arab patients in order to minimise the remarkably long delay in diagnosis.

## Figures and Tables

**Table 1:** Clinical and laboratory features seen in our Spondyloarthropathy cohort.

**Feature**	**AS**	**Other SpA**
Cohort Size	88	53
Male Proportion	71 (80.7%)	29 (54.7%)
Mean age at Symptom onset(SD)	28.4 (9.8)	33.88 (11.8)
Mean age at Diagnosis(SD)	31.9 (9.7)	37.85 (10.1)
Smoking Status		
• Never	52/76 (68.4%)	42/49 (85.7%)
• Former	4/76 (5.3%)	1/49 (2.04%)
• Current	20/76 (26.3%)	6/49 (12.3%)
HLA-B27 Positivity	58/77 (75.3%)	10/28 (35.7%)
Ethnicity		
• Indian Subcontinent	40(46.6%)	18 (34%)
• Arab	21 (23.9%)	22 (41.5%)
• Caucasian	17 (19.3%)	12 (22.6%)
• Afro Caribbean	1 (1.1%)	1 (1.9%)
• Eastern European	4 (4.5%)	0 (0%)
• East Asian	3(3.4%)	0 (0%)

AS, Ankylosing Spondylitis; Other SpA, Other Spondyloarthropathies; SD, Standard Deviation; HLA, Human Leucocyte Antigen

**Table 2:** Clinical features of the Ankylosing spondylitis and other Spondyloarthropathy patients.

**Clinical Characteristics AS** **Frequency(%)**		**Other SpA** **Frequency(%)**	**P value †**
Cohort Size	88	53	-
Inflammatory back pain	80(94)	28(53)	<0.001
Peripheral arthritis	54(61)	53(100)	<0.001
Enthesitis	14(16)	28(53)	<0.001
Uveitis	16(18)	6(11)	0.017
Psoriasis	3(3)	6(11)	0.08
IBD	2(2)	2(4)	>0.1
Last BASDAI (out of 10)*	3.37	-	-
Last Schoebers (cm)*	4.98	-	-
Sacroiliitis bilateral **∆**	70/80(88)	14/33(42)	<0.001
Sacroiliitis unilateral **∆**	7/80(9)	4/33(12)	>0.1
Bamboo spine 9(17)		0(0)	0.014

†Comparisons were performed using Fischer’s exact test *Mean Last clinic consultation BASDAI and Shoebers analysed **∆** Sacroiliitis on MRI prevalent in 93% and 36% of AS and SpA patients respectively. AS, Ankylosing Spondylitis; Other SpA, Other Spondyloarthropathies; IBD, Inflammatory Bowel Disease; BASDAI, Bath Ankylosing Spondylitis Disease Activity Index

**Table 3:** Clinical and laboratory features of Ankylosing Spondylitis and other Spondyloarthropathy patients of the three major ethnicities.

–	AS Patients			Other SpA Patients		
	**Arab Indian** **Subcontinent**	**Caucasian**	**P value†**	**Arab Indian** **Subcontinent**	**Caucasian**	**P value†**
Cohort size	21 40	17	-	12 18	22	-
Male (%)	20(95) 32(80)	12(71)	-	8(67) 9(50)	12(55)	-
Age in years at symptom onset (SD)	29.7 (9.4) 28.6 (10.2)	26.6 (8.7)	0.64	30.0 39.2 (11.7) (11.8)	31.1 (10.6)	0.05
Delay to diagnosis in years(SD)	2.89 (3.4) 3.85 (6.0)	1.87 (2.5)	0.39	6.92 1.22 (2.6) (8.7)	3.38 (7.0)	0.06
HLAB27 Positivity	10/19(53%) 28/35(80%)	14/15(93%)	0.023	2/4(50%) 2/8(25%)	5/15(33%)	0.45

†Comparisons were performed using Fisher’s exact test or a One-way ANOVA test

AS, Ankylosing Spondylitis; Other SpA, Other Spondyloarthropathies; SD, Standard Deviation; HLA, Human Leucocyte Antigen
